# The Eatwell Guide: Modelling the Health Implications of Incorporating New Sugar and Fibre Guidelines

**DOI:** 10.1371/journal.pone.0167859

**Published:** 2016-12-20

**Authors:** Linda J. Cobiac, Peter Scarborough, Asha Kaur, Mike Rayner

**Affiliations:** 1 Centre for Health Policy, School of Population and Global Health, The University of Melbourne, Melbourne, Australia; 2 British Heart Foundation Centre on Population Approaches for Non-Communicable Disease Prevention, Nuffield Department of Population Health, University of Oxford, Oxford, United Kingdom; Indiana University Bloomington, UNITED STATES

## Abstract

**Objective:**

To model population health impacts of dietary changes associated with the redevelopment of the UK food-based dietary guidelines (the ‘Eatwell Guide’).

**Method:**

Using multi-state lifetable methods, we modelled the impact of dietary changes on cardiovascular disease, diabetes and cancers over the lifetime of the current UK population. From this model, we determined change in life expectancy and disability-adjusted life years (DALYs) that could be averted.

**Results:**

Changing the average diet to that recommended in the new Eatwell Guide, without increasing total energy intake, could increase average life expectancy by 5.4 months (95% uncertainty interval: 4.7 to 6.2) for men and 4.0 months (3.4 to 4.6) for women; and avert 17.9 million (17.6 to 18.2) DALYs over the lifetime of the current population. A large proportion of the health benefits are from prevention of type 2 diabetes, with 440,000 (400,000 to 480,000) new cases prevented in men and 340,000 (310,000 to 370,000) new cases prevented in women, over the next ten years. Prevention of cardiovascular diseases and colorectal cancer is also large. However, if the diet recommended in the new Eatwell Guide is achieved with an accompanying increase in energy intake (and thus an increase in body mass index), around half the potential improvements in population health will not be realised.

**Conclusions:**

The dietary changes required to meet recommendations in the Eatwell Guide, which include eating more fruits and vegetables and less red and processed meats and dairy products, are large. However, the potential population health benefits are substantial.

## Introduction

Dietary carbohydrates are an important source of energy to support our day-to-day activities. But there is growing concern that an increasing consumption of carbohydrates in the form of free sugars, together with a decreasing consumption of higher fibre carbohydrates, is contributing to weight gain and poor health outcomes, including type 2 diabetes, cardiovascular disease and cancer [1].

Free sugars include sugars added to foods and beverages before consumption, and naturally occurring sugars such as those in honey, syrups and fruit juices, but do not include sugars incorporated within intact fruits and vegetables or sugars naturally present in milk [1]. Foods high in free sugar, such as sugar-sweetened beverages, may displace more nutrient-rich and higher fibre foods, such as fruits and vegetables, from the diet.

In the United Kingdom (UK), until recently the Government provided healthy eating advice in the form of the eatwell plate, which illustrated the different types of foods that we should eat, and the appropriate proportions required to achieve a healthy diet. Following a review of the latest evidence on carbohydrates and health, the Scientific Advisory Committee on Nutrition (SACN) advised the Government to reduce the recommended average intake of free sugars to no more than 5% of dietary energy intake and increase the recommended average intake of fibre to 30 grams per day [2]. This information has now been incorporated into the healthy eating recommendations in the form of the Eatwell Guide [3], which was launched in March 2016.

In an optimisation modelling analysis [4], we found that meeting the new dietary recommendations in the UK would require a substantial net increase in consumption of “potatoes, bread, rice, pasta and other starchy carbohydrates” (+69%) and “fruit and vegetables” (+71%), and a net reduction in consumption of “beans, pulses, fish, eggs, meat and other proteins” (-24%), “dairy and alternatives” (-29%), and “foods high in fat and sugar” (-53%). This analysis has been used for the relaunch of the UK’s food-based dietary guidelines, as the Eatwell Guide in March 2016 [3]. In this paper we present the implications of these changes in diet for the long-term health of the UK population.

## Methods

We modelled the impact of dietary changes on health of the UK population for two dietary scenarios: (1) if everyone changed from the current UK average diet to a diet that meets dietary recommendations that were in place before the SACN report of 2015 (the ‘old recommendations’ scenario); and (2) if everyone changed from the current UK average diet to a diet that meets dietary recommendations used for the new Eatwell Guide [3]. Both sets of recommendations (food based dietary guidelines) are based on achieving recommended levels of consumption of carbohydrates, free sugars, fat, saturated fat, protein, salt, fibre, fruits and vegetables, fish, and red and processed meat (Table 1). In both the Old recommendations scenario and the Eatwell Guide scenario we evaluate the population health that could be gained by changing from the current average UK diet to the recommended diet. The key differences between the old recommendations and recommendations in the new Eatwell Guide are in guidelines on free sugar intake (reduced from less than 11% of food energy to less than 5% of food energy) and fibre (increased from 23.5g to 30g per day). We evaluate the health gains achieved with both Old recommendations and new Eatwell Guide recommendations to determine the impact of the free sugar and fibre changes proposed by the latest SACN report.

**Table 1:** Current and proposed recommendations used as constraints in the optimisation modelling (after Scarborough et al [4]).

	Old recommendations	Eatwell Guide
**NUTRIENTS**		
**Energy**^1^	No increase in kcal	No increase in kcal
**Carbohydrates**	≥50% of energy	≥50% of energy
**Free sugars**	≤11% energy	≤5% energy
**Fat**	≤35% energy	≤35% energy
**Saturated fat**	≤11% energy	≤11% energy
**Protein**	≥14.5 & ≤15.5% of energy	≥14.5 & ≤15.5% of energy
**Salt**	≤ 2363 mg sodium	≤ 6g/2363 mg sodium
**Fibre**	≥ 23.5g AOAC	≥30g (AOAC)^2^
**FOODS**		
**Fruits and vegetables**^3^	≥5 portions a day	≥5 portions a day
**Fish**	≥ 2 portions a week (2*20g a day), one of which should be oily	≥ 2 portions a week (2*20g a day), one of which should be oily
**Red and processed meat**	≤70g/day	≤70g/day

NB. AOAC: Association of Official Analytical Chemists method for total dietary fibre analysis.

^1^ Energy from foods and drinks, excluding alcohol.

^2^ Equivalent 18g non-starch polysaccharide fibre

^3^ Includes a maximum of: 1 portion of fruit juice; 1 portion of beans; 2 portions of smoothie. (Portion sizes: 30g dried fruit; 150mL fruit juice; 150mL smoothie; 80g all other fruits & vegetables)

In a previous paper we have described how we used optimisation to identify the diet for each scenario that is minimally changed from the current UK diet, but meets recommended intakes of nutrients and foods, and does not lead to an increase in total energy [4]. For this paper, we also re-ran the analyses to examine the impact on population health if the constraint on total energy intake is removed (i.e. there is no upper bound on energy intake). Changes in dietary intake are shown in Table 2. For the health modelling, we determined changes in dietary intake separately by age and sex, by applying the percentage change in intake from the optimisation, which was calculated for the total adult population aged 19+ years, to age- and sex-specific baseline values of intake from the National Diet and Nutrition Survey 2008–2012 [5].

**Table 2:** Change in intake of selected food groups and energy intake.

	Current average intake, g/d	Change in intake*, g/day			
		Energy intake constrained		No energy intake constraint	
		Old recommendations	Eatwell Guide	Old recommendations	Eatwell Guide
**Fruit and vegetables**	**342**	**↑ 58**	**↑ 184**	**↑ 57**	**↑ 100**
Fruit	102	**↑** 13	**↑** 103	**↑** 17	**↑** 27
Fruit juice	63	**↑** 1	**↓** -31	**↑** 2	**↓** -8
Dried fruit	4.6	**↑** 1	**↑** 3.3	**↑** 1.3	**↑** 1
Vegetables	171	**↑** 41	**↑** 113	**↑** 37	**↑** 78
**Potatoes, bread, rice, pasta and other starchy carbohydrates**	**281**	**↑ 54**	**↑ 192**	**↑ 56**	**↑ 156**
Brown/wholemeal bread	33	**↑** 15	**↑** 50	**↑** 11	**↑** 37
White bread	49	**↑** 2	**↑** 19	**↑** 10	**↑** 24
Rice	27	**↑** 1	**↑** 1	**↑** 3	**↑** 7
Pasta	25	**↑** 2	**↑** 10	**↑** 3	**↑** 11
Potatoes	91	**↑** 14	**↑** 82	**↑** 15	**↑** 43
Cereals	8.3	**↑** 2.7	**↑** 2.7	**↑** 3.7	**↑** 13.7
Breakfast cereals, high fibre	20	**↑** 8	**↑** 32	**↑** 7	**↑** 18
Breakfast cereals, not high fibre	5.6	**↑** 2.5	**↓** -0.5	**↑** 4.4	**↑** 0.4
**Dairy and alternatives**	**221**	**↓ -24**	**↓ -48**	**↓ -15**	**↓ -27**
Milk	170	**↓** -7	**↓** -15	**↓** -3	**↓** -8
Cheese	17	**↓** -12.8	**↓** -14.4	**↓** -10	**↓** -16
Yoghurt	27	**↓** -1	**↓** -15	**↔** 0	**↓** -3
**Beans, pulses, fish, eggs, meat and other proteins**	**212**	**↓ -28**	**↓ -52**	**↑ 3**	**↑ 7**
Red meat**	35	**↓** -22	**↓** -27.3	**↓** -10	**↓** -16
Processed meat	33	**↓** -16	**↓** -25.8	**↓** -12	**↓** -22
White meat***	35	**↓** -11	**↓** -30	**↓** -4	**↓** -8
Oily fish	8.7	**↑** 11.3	**↑** 29.3	**↑** 11.3	**↑** 11.3
Whitefish	16	**↑** 4	**↑** 7	**↑** 4	**↑** 4
Beans, pulses and other legumes	14	**↑** 11	**↑** 12	**↑** 9	**↑** 19
Nuts	2.7	**↑** 3.4	**↓** -0.1	**↑** 5.3	**↑** 12.3
**Foods high in fat and sugar**	**216**	**↓ -3**	**↓ -113**	**↑ 23**	**↓ -58**
Sugar sweetened beverages	120	**↓** -1	**↓** -61	**↔** 0	**↓** -21
Low calorie beverages	85	**↔** 0	**↓** -2	**↔** 0	**↓** -1
Cakes, confectionary and biscuits	71	**↑** 5	**↓** -40	**↑** 18	**↓** -49
Crisps and savoury snacks	6.1	**↑** 3.9	**↓** -0.1	**↑** 5.9	**↑** 12.9
Oils and spreads	14	**↓** -9.6	**↓** -8.5	**↓** -4	**↓** -7
**Energy (kcal)**	**1711**	**1711**	**1711**	**1926**	**1984**

*Change in comparison to the current average intake.

**Beef, lamb and pork,

***Chicken and other poultry

We used a multi-state lifetable model, PRIMEtime (S1 File), to simulate the impact of different dietary scenarios on diet-related diseases in the UK adult population. We modelled the health of the population forward in time from 2014, assuming that the population could change their diet immediately and that the changes would be sustained.

PRIMEtime simulates the current population over time until everyone has died, taking account of changes in incidence, prevalence and mortality of lifestyle risk factor-related diseases. Changes in diet have an impact on disease, either directly (e.g. fibre intake and colorectal cancer) or via intermediate variables (blood pressure, cholesterol and body mass index). Our modelling of these relationships was based on evidence of significant effects in meta-analyses of epidemiological studies that had examined associations between diet and disease. The diseases influenced by changes in intake of foods (e.g. fruits, vegetables, red and processed meats) and nutrients (e.g. fibre and sodium) include coronary heart disease (CHD), stroke, type 2 diabetes and cancer of the breast, colorectum, lung, and stomach. Changes in energy intake (which affect body mass) additionally influence risk of cirrhosis and cancers of the pancreas, kidney and liver. The risk factors and diseases included in the PRIMEtime modelling of the dietary scenarios, and data sources used in the analyses, are shown in Table 3.

**Table 3:** Dietary and related metabolic risk factors, population exposure to risks and disease outcomes included in PRIMEtime.

Risk factor	Exposure parameters	Outcomes
Fruit intake	Mean (SD) g/day for consumers and % consuming <1 fruit portion daily from NDNS. Theoretical ideal: 300 (30) g/day [6]	CHD [7]; Stroke [8]; Lung cancer [9]
Vegetable intake	Mean (SD) g/day for consumers and % consuming <1 vegetable portion daily from NDNS. Theoretical ideal: 400 (30) g/day [6]	CHD [7]; Lung cancer [9]
Fibre intake	Mean (SD) g/day from NDNS. Theoretical ideal: 30 (3) g/day [6]	Breast cancer [10]; Colorectal cancer [11]; Stomach cancer [12]
Fibre intake (cereal only)	Mean (SD) g/day from NDNS	CHD [13]
Red meat intake	Mean (SD) g/day from NDNS. Theoretical ideal: 100 (10) g/week [6]	Colorectal cancer [14]; Stomach cancer [15]; Type 2 diabetes [16]
Processed meat intake	Mean (SD) g/day from NDNS. Theoretical ideal: 0 g/day [6]	Colorectal cancer [14]; Type 2 diabetes [16]
Sodium	mmol/24hr	Blood pressure [17]
Free sugars	% of total energy	Total cholesterol [18]
Total fat	% of total energy	Total cholesterol [19]
Saturated fat	% of total energy	Total cholesterol [19]
Monounsaturated fat	% of total energy	Total cholesterol [19]
Polyunsaturated fat	% of total energy	Total cholesterol [19]
Dietary cholesterol	mg/day	Total cholesterol [19]
Total energy	kJ/day	BMI [20]
BMI	Mean (SD) kg/m2 from NDNS. Theoretical ideal: 21 (1) kg/m2 [21]	CHD [22]; Stroke [22]; Diabetes [22]; Cirrhosis [22]; Colorectal cancer [22]; Kidney cancer [22]; Breast cancer [22]; Pancreas cancer [23]; Liver cancer [22]
Blood pressure	Mean (SD) mmHg from NDNS. Theoretical ideal: 115 (6) mmHg [21]	CHD [24]; Stroke [24]
Total serum cholesterol	Mean (SD) mmol/L from NDNS. Theoretical ideal: 3.8 (0.6) mmol/L [21]	CHD [25]; Stroke [25]

NB. CHD: coronary heart disease; SD: standard deviation; NDNS: National Diet and Nutrition Survey [5]; BMI: body mass index

To calculate the impact of dietary changes on disease incidence, we first calculated the effects of dietary changes on intermediate variables. This included calculation of changes in:

- systolic blood pressure from changes in salt intake, using effect estimates from meta-analysis of salt reduction randomised trials [17]
- total serum cholesterol from changes in fats (total, saturated, monounsaturated and polyunsaturated) and dietary cholesterol, using effect estimates from meta-analysis of metabolic ward studies of solid food diets in healthy volunteers [19];
- total serum cholesterol from changes in free sugars, using effect estimates from meta-analysis of trials of free sugar reduction [18]; and
- body mass index from changes in total energy intake, using energy balance equations [20] and measurements of height from the National Diet and Nutrition Survey [5].

We then quantified the impact of each dietary or metabolic risk on disease, using the population impact fraction (PIF) equation to determine the change in probability of disease incidence, based on change in prevalence of the risk factor and relative risk or the disease:

$$\text{PIF }=\;\frac{\int_{a}^{b}\text{p }\left(x\right)\text{ RR }\left(x\right)\;dx\;-\;\int_{a}^{b}\text{p}\prime \;\left(x\right)\text{ RR }\left(x\right)\;dx}{\int_{a}^{b}\text{p }\left(x\right)\text{ RR}\left(x\right)\;dx}$$

where:

p(*x*) is the current prevalence distribution of a risk factor;

p′(*x*) is the prevalence distribution of the risk factor after the diet is changed;

RR(*x*) is the distribution function for the relative risk of disease; and

*a* and *b* are the lower and upper bounds of the integration.

The baseline prevalence of dietary and metabolic risks, including intake of fruits, vegetables, fibre, red meat, processed meat, sodium, total energy, free sugars, body mass index, blood pressure and total cholesterol, were derived from the National Diet and Nutrition Survey 2008–2012 [5]. The ideal population levels of exposure to these risk factors are reflected by the theoretical risk exposure distributions presented in Table 3. These values were derived from a combination of epidemiological evidence and expert opinion for Global Burden of Disease analyses [6].

We selected relative risks for dietary parameters (e.g. fibre, fruits, vegetables, red meats and processed meats) that had been adjusted for energy. Where a disease was influenced by more than one dietary or metabolic risk factor, we calculated the combined impact multiplicatively, adjusting for risk-disease pathways that are not independent using mediation factors estimated in the Global Burden of Disease study [26], and selecting risks to avoid double-counting of effects (e.g. risk of CHD from cereal fibre rather than total fibre, to avoid overlap with risk of CHD associated with fruit and vegetable intake). This primarily influenced calculation of CHD and stroke effects, which are associated with changes in many dietary and behavioural risks (e.g. CHD risk is influenced by fruit and vegetable intake, fibre intake, blood pressure, cholesterol and BMI; and the effects of BMI may be partially mediated by changes in blood pressure). Further details about the disease risk adjustments can be found in S1 File.

To determine the impact of dietary changes on population health measures, we ran the PRIMEtime model both with and without dietary changes. The baseline scenario reflected ‘business-as-usual’, where current trends in incidence and case fatality from diseases (e.g. due to changing prevalence of smoking, increased availability of disease treatments, etc.) are assumed to continue into the future. The scenarios simulated the effect of dietary changes on disease, over-and-above current trends. From the difference between the baseline and simulations, we determined the impact on population life expectancy and on number of disability-adjusted life years (DALYs) that would be averted. DALYs are a summary measure of health that reflects impact on both morbidity and mortality from disease. They are calculated in the PRIMEtime model from the number of years of life that are lived by the population, adjusted for time spent in ill-health (i.e. with ‘disability’ from diseases). The disability adjustment is determined from disease disability weights measured in the Global Burden of Disease study [27].

In addition to the health impact, we also estimated the contribution of the dietary risk factors to the overall health gain, by eliminating each risk factor causal pathway in turn. For each scenario, we estimated the impact of each risk factor from the increase or decrease in total DALYs when the risk factor is eliminated.

We determined uncertainty (95% uncertainty intervals) for our main outcome measures (cases of disease prevented, DALYs averted and change in life expectancy) using Monte Carlo analysis. A table of the uncertainty around modelling input parameters, as well as further information about the design of the PRIMEtime model and sources of epidemiological data, can be found in S1 File.

## Results

### Total health impact

**Fig 1:**
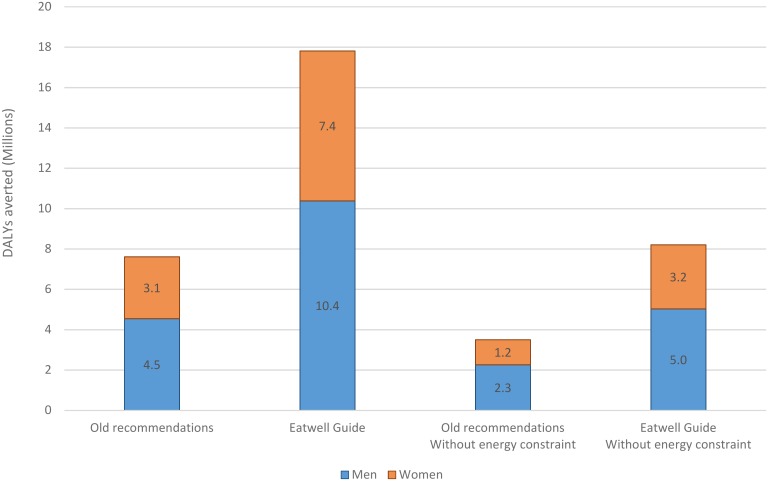
DALYs averted overt the lifetime of the UK population.

There are substantial health benefits to be gained if UK adults can change to a diet that meets recommended levels of foods and nutrients without increasing total energy intake (Fig 1). Changing to a diet that meets old recommendations could avert 7.5 million (95% uncertainty interval (UI): 7.2 to 7.8) DALYs over the lifetime of the current population, but this would more than double to 17.9 million (95% UI: 17.6 to 18.2) DALYs with the stronger free sugar and fibre recommendations in the Eatwell Guide (Table 4).

**Table 4:** The average increase in population life expectancy and health gain that could be achieved.

	Old recommendations	Eatwell Guide	Old recommendations Without energy constraint	Eatwell Guide Without energy constraint
**Increase in life expectancy (months)**				
Men	2.3 (2.0 to 2.6)	5.4 (4.7 to 6.2)	1.3 (1.0 to 1.6)	2.8 (2.3 to 3.4)
Women	1.6 (1.3 to 1.8)	4.0 (3.4 to 4.6)	0.9 (0.7 to 1.2)	2.0 (1.5 to 2.5)
**DALYs averted (millions)**				
Men	4.6 (4.3 to 4.8)	10.4 (10.0 to 10.8)	2.3 (2.1 to 2.5)	5.0 (4.7 to 5.4)
Women	3.1 (2.9 to 3.2)	7.4 (7.1 to 7.7)	1.2 (1.1 to 1.4)	3.2 (2.9 to 3.4)

NB. DALYs: Disability-adjusted life years. Values are means and 95% uncertainty intervals.

If everyone followed the old dietary recommendations, average life expectancy of the population would increase by 2.3 (2.0 to 2.6) months for men and 1.6 (1.3 to 1.8) months for women, but average improvements in life expectancy would be greater if everyone followed the dietary recommendations in the Eatwell Guide, increasing by 5.4 (4.7 to 6.2) months for men and 4.0 (3.4 to 4.6) months for women (Table 4). The potential improvements in health are greater for men than women because men, on average, have a poorer quality of diet to begin with (e.g. lower in fruits and vegetables, higher intake of red and processed meats).

Without a constraint on energy intake the potential health gains from changing to a diet that meets either old recommendations or recommendations in the new Eatwell Guide, are much smaller. There would be 52% fewer DALYs averted for men and 57% fewer DALYs averted for women, under both dietary recommendation scenarios. Potential improvements in life expectancy are around 40% lower for a diet that meets old recommendations, and around 50% lower for a diet that meets recommendations in the new Eatwell Guide, when energy intake is allowed to increase.

### Disease impacts

When the total energy of the diet is prevented from increasing, a large proportion of the health benefits are from prevention of type 2 diabetes (Table 5). Achieving a diet that meets Eatwell Guide recommendations would reduce new cases of diabetes by 440,000 (400,000 to 480,000) in men over the next ten years, and by 340,000 (310,000 to 370,000) in women over the next ten years. The prevention of cardiovascular diseases, both directly and through prevention of type 2 diabetes (an important cardiovascular risk factor) and prevention of colorectal cancer is also large. A smaller, but still substantial, number of cases of stomach, lung and breast cancer would also be prevented. The pattern of disease outcomes is similar with a diet that meets old recommendations, but substantially fewer cases of disease would be averted (ranging from 38% fewer cases of diabetes up to 79% fewer cases of stroke).

**Table 5:** New cases of disease that are averted or delayed in the first 10 years.

		Old recommendations	Eatwell Guide	Old recommendations without energy constraint	Eatwell Guide without energy constraint
**Men**	**CHD**	59,000 (54,000 to 64,000)	170,000 (160,000 to 180,000)	40,000 (36,000 to 45,000)	100,000 (93,000 to 110,000)
	**Stroke**	16,000 (15,000 to 18,000)	80,000 (71,000 to 89,000)	8,100 (5,800 to 10,000)	14,000 (11,000 to 17,000)
	**Diabetes**	280,000 (250,000 to 310,000)	440,000 (400,000 to 480,000)	44,000 (15,000 to 71,000)	150,000 (110,000 to 200,000)
	**Breast cancer**	-	-	-	-
	**Colorectal cancer**	64,000 (58,000 to 70,000)	110,000 (99,000 to 110,000)	39,000 (34,000 to 44,000)	73,000 (65,000 to 82,000)
	**Lung cancer**	13,000 (11,000 to 15,000)	49,000 (42,000 to 55,000)	14,000 (12,000 to 16,000)	24,000 (20,000 to 28,000)
	**Stomach cancer**	16,000 (15,000 to 18,000)	28,000 (26,000 to 30,000)	16,000 (14,000 to 17,000)	27,000 (24,000 to 29,000)
	**Pancreas cancer**	-51 (-55 to -47)*	-140 (-150 to -130)*	-7,600 (-8,300 to -6,800)	-9,800 (-11,000 to -8,800)
	**Kidney cancer**	-58 (-62 to -54)*	-160 (-170 to -150)*	-850 (-950 to -750)	-1,100 (-1,200 to -990)
	**Liver cancer**	-59 (-64 to -55)*	-160 (-170 to -150)*	-2,500 (-2,800 to -2,200)	-3,300 (-3,600 to -2,900)
	**Cirrhosis**	-38 (-40 to -35)*	-100 (-110 to -95)*	-2,300 (-2,600 to -1,900)	-2,900 (-3,400 to -2,500)
**Women**	**CHD**	32,000 (29,000 to 34,000)	94,000 (87,000 to 100,000)	20,000 (17,000 to 23,000)	54,000 (48,000 to 59,000)
	**Stroke**	17,000 (15,000 to 19,000)	84,000 (74,000 to 94,000)	8,600 (6,000 to 11,000)	14,000 (11,000 to 18,000)
	**Diabetes**	200,000 (180,000 to 220,000)	340,000 (310,000 to 370,000)	-22,000 (-43,000 to 44)	44,000 (9,700 to 80,000)
	**Breast cancer**	19,000 (14,000 to 23,000)	40,000 (31,000 to 49,000)	12,000 (7,600 to 16,000)	32,000 (23,000 to 41,000)
	**Colorectal cancer**	33,000 (30,000 to 36,000)	60,000 (55,000 to 65,000)	24,000 (21,000 to 26,000)	44,000 (39,000 to 49,000)
	**Lung cancer**	8,700 (7,200 to 10,000)	33,000 (28,000 to 37,000)	9,200 (7,900 to 11,000)	16,000 (13,000 to 19,000)
	**Stomach cancer**	8,000 (7,200 to 8,900)	15,000 (14,000 to 16,000)	7,900 (7,000 to 8,700)	14,000 (13,000 to 16,000)
	**Pancreas cancer**	-24 (-25 to -22)*	-71 (-75 to -66)*	-5,100 (-5,600 to -4,600)	-6,600 (-7,200 to -5,900)
	**Kidney cancer**	-3.8 (-4.0 to -3.5)*	-11.0 (-12.0 to -10.0)*	-210.0 (-240.0 to -190.0)	-280.0 (-310.0 to -250.0)
	**Liver cancer**	-19 (-20 to -18)*	-57 (-61 to -53)*	-2,000 (-2,200 to -1,900)	-2,600 (-2,800 to -2,400)
	**Cirrhosis**	-59 (-62 to -55)*	-170 (-180 to -160)*	-8,700 (-9,900 to -7,500)	-11,000 (-13,000 to -9,600)

NB. CHD: coronary heart disease. Values are means and 95% uncertainty intervals.

* These numbers increase because people are living longer due to other diseases being prevented (i.e. there is no direct effect from a change in energy and BMI)

Without any constraints on energy in the diet optimisation, energy intake increases by 13% when the diet is optimised to meet the old recommendations and increases by 16% when the diet is optimised to meet the recommendations in the Eatwell Guide. In our modelling, this leads to an increase in BMI and related diseases, including diabetes, cardiovascular disease, cirrhosis and a range of cancers. While these effects are countered by beneficial effects from increases in intake of fruits, vegetables and fibre, and a reduction in intake of unhealthy fats, red and processed meats, the net effect would be an increase in future cases of diabetes for men and women with a diet that meets old recommendations and for women (but not men) with a diet that meets recommendations in the Eatwell Guide. There would also be an increase in cases of cirrhosis and cancers of the pancreas, kidney and liver, due to the rise in BMI levels.

### Mediating risk factors

**Fig 2:**
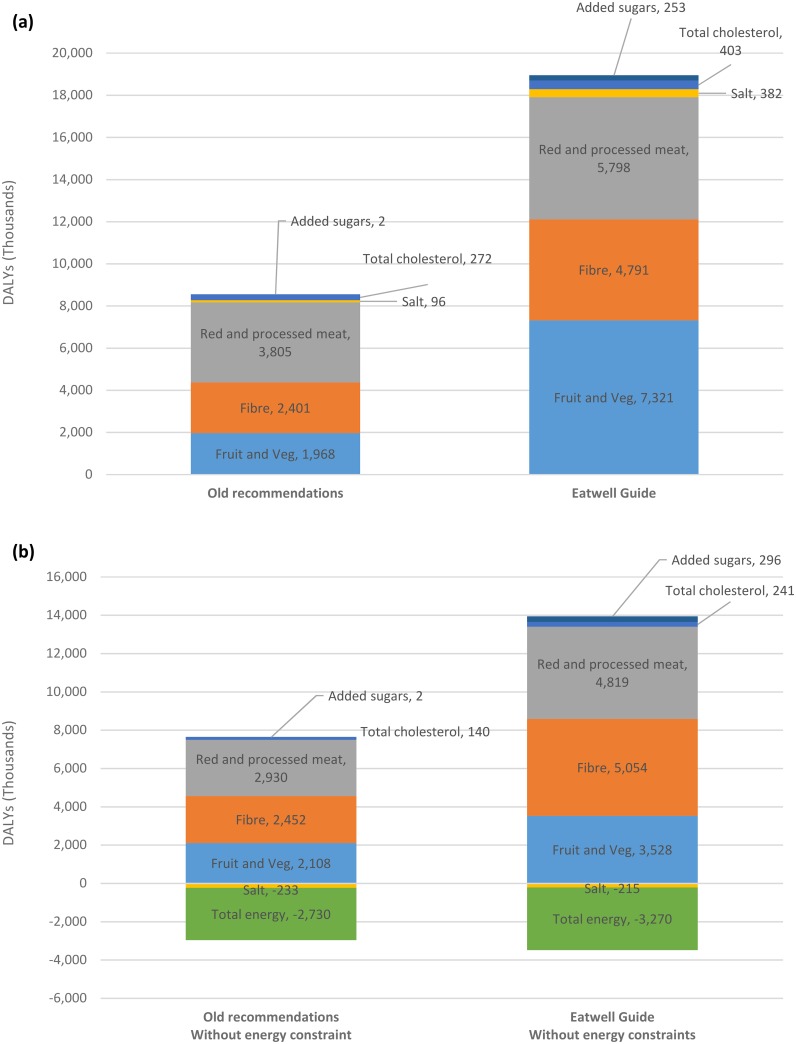
The contribution of modelled risk factors to the net gain in health when: (a) total energy intake is constrained; and (b) energy intake is not constrained. (NB. each value reflects the change in total DALYs if the risk factor is eliminated from the analyses).

Fig 2 shows the contribution of modelled risk factors to the net gain in health. The negative values for salt and total energy in Fig 2(b) indicate that these risks are associated with a health loss in the modelled analyses. Note that the sum of the DALYs in each scenario is not exactly equivalent to the total DALYs averted by the scenario (Table 4) because of the assumption that risk factors are multiplicative (i.e. each additional risk factor only reduces the remaining health burden).

When total energy of the diet is not allowed to increase, the majority of health gain is from reductions in red and processed meats, and increases in fibre, fruts and vegetables, with changes in other dietary risks (free sugars, total cholesterol and sodium) having only a minor impact. When total energy of the diet is allowed to increase, the changes in red and processed meats, fibre and fuits and vegetables are still responsible for the majority of health gain, but there is also a substantial health loss associated with the increase in energy intake.

Although the only differences between the Old recommendations and Eatwell Guide scenarios are the stricter free sugar and fibre constraints in the optimisation model, only around 25% of the added health gains are actually mediated by the free sugar (2%) and fibre (23%) risk factors in the health model, when energy is constrained. Without any constraint on energy, around 50% of the added health gains are mediated by the free sugar (5%) and fibre (45%) risk factors. The remaining proportion of the health gain is primarily mediated by the fruit and vegetable and red and processed meat risk factors. The changes in consumption of fruits and vegetables and red and processed meats come about because of the shift in consumption patterns produced by stricter sugar and fibre guidelines. As can be seen in Table 2, for example, the high free sugar products (e.g. sugar sweetened beverages, fruit juices, cakes, confectionary and biscuits) and lower fibre products (e.g. breakfast cereals, not high fibre) are ‘replaced’ in the diet by more fruits and vegetables and higher fibre carbohydrates. In addition, when energy intake is constrained (i.e. is not allowed to increase), the overall increase in carbohydrate-rich foods is associated with a decrease in intake of protein-rich foods (e.g. red and processed meats).

## Discussion

The potential benefits to population health from the updates to dietary recommendations in the Eatwell Guide are substantial. The strengthening of sugar and fibre recommendations more than doubles the health that could potentially be gained if everyone were to follow dietary recommendations without increasing energy intake. It is important that people are encouraged to improve diet quality, by shifting between food groups (e.g. more fruits and vegetables, and less meat), without increasing overall energy intake of the diet, which can have substantial impact on obesity and related diseases, lessening the improvements in health. The results show how big changes in fruit and vegetables and meat consumption that are recommended in the Eatwell Guide could translate into big improvements in health when compared to average consumption levels in the current UK diet.

If average energy intake of the diet does not increase, the population health benefits from prevention of type 2 diabetes are particularly large. As in many countries, the prevalence of type 2 diabetes in the UK has been increasing [28], and treatment of diabetes and its complications is now responsible for around 10% of the National Health Service budget [29]. Helping people to achieve a healthier diet could be crucial to addressing this growing burden.

Although SACN has published energy recommendations, these were not applied by Scarborough et al [4] in deriving the new plate proportions (e.g. 39% fruit and vegetables, 8% dairy and alternatives, etc.) for the Eatwell Guide. This is because our modelling is based on National Diet and Nutrition Survey data, which is (as with other dietary surveys) suffers from under-reporting. The average energy intake found in the data used for this study was 1711kcal/d. To apply the SACN energy recommendations would mean incorporating constraints which would force consumption of energy to increase, which is incompatible with UK Government aims to reduce obesity.

For modelling health outcomes in this paper, the optimisation modelling was re-run without the constraint on energy, to illustrate the potential magnitude of effect on BMI and related diseases if energy intake was allowed to vary freely. We found that without the energy constraint, average adult energy intake increased by 16% to meet the dietary recommendation in the new Eatwell Guide. In our modelling, this led to an increase in BMI (assuming no increase in energy expenditure) and a loss of health from BMI-related diseases. However, an increase in physical activity could potentially counter these health losses.

It should also be noted that the derivation of the modelled diets by Scarborough et al [4] used baseline consumption data from the NDNS. The average energy intake estimated in this survey (1711kcal) was low in comparison to estimated energy requirements (2000kcal for adult women and 2500 kcal for adult men [3]), which is most likely due to under-reporting in the survey. It is possible that this has led to either an under- or over-estimate of the health impacts of dietary changes, depending on whether the constraints in the optimisation model encourage an increase in consumption (e.g. fruits and vegetables) or a decrease in consumption (e.g. saturated fats), and on whether some foods (e.g. unhealthy cakes, confectionary and biscuits) are more likely to be under-reported than other healthier foods (e.g. fruits and vegetables).

The model simulates what would happen to the health of the current population if everyone was to change to a healthier diet. It takes current trends in the incidence and case fatality of the dietary-related diseases into account, and disease risks have been selected and adjusted to avoid double-counting of health effects from dietary changes. There are, however, still ambiguities in the causal pathways between diet and disease outcomes in the epidemiological literature. Ideally, all of the relative risk parameters that have been included in the PRIMEtime model would be mutually adjusted for each other, but this is not possible when using parameters sourced from published systematic reviews. Similarly, all of the relative risk parameters included in PRIMEtime have been taken from meta-analyses of prospective cohort studies. Although these observational studies have been adjusted for potentially confounding factors (e.g. age, sex, smoking, social class) it is not possible to remove the possibility of residual confounding either due to missing explanatory variables, or poorly measured explanatory variables.

In addition, there is uncertainty in model parameters, such as future trends in diseases and current measurements of diet, that we have not included. There is also uncertainty in the structural decisions we have made in the model design. In using a proportional multi-state lifetable model of diseases, for example, we assume that the disease processes are independent. While we do address links between diabetes prevalence and cardiovascular disease outcomes, where we have good evidence of risks, it is likely that there are other interdependencies that we do not capture (e.g. a slight increase in risk of cancers in those with cardiovascular disease). It is very likely therefore that there is greater uncertainty in our results than we have been able to quantify in the modelling.

The recent analyses of disease burden in the UK [26] and England [30], both identified dietary risks as the leading contributor to DALYs in 2013. These burden of disease DALYs, which reflect the total burden in one year, are not directly comparable to the DALYs calculated in our multi-state lifetable models, which capture the DALYs that would be averted over the future lifetime of the 2014 population. Nevertheless, as in our study, the researchers found that men stood to gain a greater improvement in health than women, from improving diet, and that the health would be gained from prevention of a similar range of diseases. Similar to our analyses, low intake of fruits, vegetables and fibre and high intake of red and processed meats were responsbile for a large proportion of the diet-related DALYs. However, the burden of disease analyses showed a much higher proportion of DALYs attributable to high sodium intake (i.e. that more health could be gained from sodium reduction than we have modelled).

This difference in outcomes relating to sodium is most likely due to a difference in the optimal intake of salt that is modelled in the two studies. The old dietary recommendation for average salt intake in England is ≤6g/day of salt, and this has not changed with the new Eatwell Guide. After more than a decade of intervention to lower salt consumption in the UK, the average salt intake, as measured from dietary intake in the NDNS [5], is relatively close to this target amount (2920 mg/day for men and 1831 mg/day for women). The optimal diets we have modelled, therefore, require little change in salt intake. Burden of disease analyses, however, model a reduction in salt to a theoretical minimum risk exposure level between 1 and 5 g/day (with a uniform probability of being within this range) [26], which is considerably less than the UK guidelines, and leads to a greater health benefit than we have modelled. There is uncertainty about the cardiovascular disease risk at lower levels of salt intake, with some studies finding a ‘J’ or ‘U’ shaped dose-response relationship and recommending against lowering salt intake too low [31], while other researchers argue that the association at low levels of salt intake is not causal [32].

Despite the differences in outcomes around sodium, the implications of our dietary modelling and the burden of disease analyses are broadly similar. However, there is an important difference in the way that the counterfactual diet has been determined. In the burden of disease studies, the counterfactual diet is an ‘ideal’ in which everyone achieves the theoretical minimum risk exposure level (e.g. red meat consumption between 11.4g and 17.1g per day, and fibre consumption between 28g and 32g per day, and sodium consumption between 1g and 5g per day, etc.). In our population health modelling analyses, however, the counterfactual diet that is input to the model has been derived using optimisation, which identifies a diet that is minimally changed from current food and drink consumption, but meets recommended levels of intake for nutrients (e.g. carbohydrates, protein, fats, salt) and foods (e.g. fruits and vegetables, red meat, fish). In some cases, these UK recommendations are different to targets used in other analyses (e.g. UK recommendation for total red and processed meat intake of ≤70g/day compared with global buden of disease targets of 100g/week for red meat and 0g intake for processed meat). Unlike the burden of disease study, the optimisation analyses can identify a diet that meets recommendations, and this diet may include changes in average consumption of foods that are currently within UK recommended ranges (e.g. red and processed meats) or for which there are no current recommendations (e.g. dairy products). The optimisation does not, however, take people’s preferences into account, such as the propensity to eat certain foods together (e.g. cereal with milk) or likelihood of substituting one food for another (e.g. regular milk for low fat milk).

To achieve the health gains that we have modelled, the UK population would need to increase consumption of fruits and vegetables, carbohydrate-based foods, fish and legumes, while reducing consumption of red, processed and white meats, dairy products and foods high in fat and sugar. Our previous study found that the impact on total food and drink costs for the individual would on average be insignificant, actually reducing slightly from 2016£6.02 (95% confidence interval: £5.96 to £6.08) to £5.99 (£5.93 to £6.05). However there would be economic implications for the meat and dairy industries due to reduced demand for their products. While agriculture adds just 0.7% to GDP in the UK [33], meat and dairy commodities are responsible for two-thirds of that value [34].

From a global perspective, there may be benefits from reduced farming of animals for food production. Animal-based products generally contribute more to greenhouse gas (GHG) emissions than plant-based products [35, 36] and consumption of more plant-based diets are associated with lower GHG emissions [37]. Optimisation studies in the UK [38], France [39] and New Zealand [40] have found that achieving a diet that meets current dietary recommendations and reduces GHG emissions requires a reduction in consumption of meat and dairy, consistent with the Eatwell Guide diet that we have modelled.

Under the Climate Change Act 2008, the UK Government has committed to achieving an 80% reduction from 1990 levels of GHG emissions by 2050 [41]. While the modelled dietary changes are consistent with climate change policy in the UK, further work is needed to quantify the magnitude of the potential GHG emissions benefits associated with the Eatwell Guide.

In addition, while we have shown that population health could improve substantially by adhering to the dietary recommendations in the Eatwell Guide, further research is needed to identify interventions that will help people change their dietary choices. Research in countries such as Australia [42, 43] and New Zealand [44] suggests that population approaches to dietary intervention, such as taxes and regulation, are likely to be more effective and cost-effective than individually-targeted approaches, such as guidance from a dietitian or general practitioner. However, the cost-effectiveness of dietary interventions has not yet been comprehensively evaluated in the UK.

## Supporting Information

S1 File The PRIMEtime model. (PDF)

## References

[1] WHO. Guideline: Sugars intake for adults and children. Geneva: World Health Organization, 2015. 25905159

[2] Scientific Advisory Committee on Nutrition. Carbohydrates and Health. UK: Public Health England, 2015.

[3] Public Health England in association with the Welsh Government FSSatFSAiNI. The Eatwell Guide. Public Health England, 2016 Contract No.: 9 August 2016.

[4] Scarborough P, Kaur A, Cobiac L, Owens P, Parlesak A, Sweeney K, et al. The Eatwell Guide: modelling the dietary and cost implications of incorporating new sugar and fibre guidelines. Under review. 10.1136/bmjopen-2016-013182 PMC5223664 28003292

[5] NatCen Social Research, MRC Human Nutrition Research, University College London Medical School. National Diet and Nutrition Survey Years 1–4, 2008/09-2011/12. UK Data Service, 2015.

[6] Lim S, Vos T, Flaxman A, Danaei G, Shibuya K, Lopez A, et al The burden of disease and injury attributable to 67 risk factors and risk factor clusters in 21 regions 1990–2010: a systematic analysis. The Lancet. 2013;380(9859):2224–60. 10.1016/S0140-6736(12)61766-8 PMC4156511 23245609

[7] Dauchet L, Amouyel P, Hercberg S, Dallongeville J. Fruit and vegetable consumption and risk of coronary heart disease: a meta-analysis of cohort studies. The Journal of nutrition. 2006;136(10):2588–93. 16988131 10.1093/jn/136.10.2588

[8] Dauchet L, Amouyel P, Dallongeville J. Fruit and vegetable consumption and risk of stroke a meta-analysis of cohort studies. Neurology. 2005;65(8):1193–7. 10.1212/01.wnl.0000180600.09719.53 16247045

[9] Vieira AR, Abar L, Vingeliene S, Chan D, Aune D, Navarro-Rosenblatt D, et al Fruits, vegetables and lung cancer risk: a systematic review and meta-analysis. Ann Oncol. 2016;27(1):81–96. 10.1093/annonc/mdv381 26371287

[10] Aune D, Chan D, Greenwood D, Vieira A, Rosenblatt DN, Vieira R, et al Dietary fiber and breast cancer risk: a systematic review and meta-analysis of prospective studies. Ann Oncol. 2012:mdr589. 10.1093/annonc/mdr589 22234738

[11] Aune D, Chan DS, Lau R, Vieira R, Greenwood DC, Kampman E, et al Dietary fibre, whole grains, and risk of colorectal cancer: systematic review and dose-response meta-analysis of prospective studies. BMJ. 2011;343. 10.1136/bmj.d6617 PMC3213242 22074852

[12] Zhang Z, Xu G, Ma M, Yang J, Liu X. Dietary fiber intake reduces risk for gastric cancer: a meta-analysis. Gastroenterology. 2013;145(1):113–20. e3. 10.1053/j.gastro.2013.04.001 23567349

[13] Threapleton DE, Greenwood DC, Evans CE, Cleghorn CL, Nykjaer C, Woodhead C, et al Dietary fibre intake and risk of cardiovascular disease: systematic review and meta-analysis. BMJ. 2013;347:f6879 10.1136/bmj.f6879 24355537 PMC3898422

[14] Aune D, Chan DS, Vieira AR, Rosenblatt DAN, Vieira R, Greenwood DC, et al Red and processed meat intake and risk of colorectal adenomas: a systematic review and meta-analysis of epidemiological studies. Cancer Causes Control. 2013;24(4):611–27. 10.1007/s10552-012-0139-z 23380943

[15] Song P, Lu M, Yin Q, Wu L, Zhang D, Fu B, et al Red meat consumption and stomach cancer risk: a meta-analysis. J Cancer Res Clin Oncol. 2014;140(6):979–92. 10.1007/s00432-014-1637-z 24682372 PMC11823590

[16] Aune D, Ursin G, Veierød M. Meat consumption and the risk of type 2 diabetes: a systematic review and meta-analysis of cohort studies. Diabetologia. 2009;52(11):2277–87. 10.1007/s00125-009-1481-x 19662376

[17] He FJ, Li J, MacGregor GA. Effect of longer term modest salt reduction on blood pressure: Cochrane systematic review and meta-analysis of randomised trials. BMJ. 2013;346. 10.1136/bmj.f1325 23558162

[18] Te Morenga LA, Howatson AJ, Jones RM, Mann J. Dietary sugars and cardiometabolic risk: systematic review and meta-analyses of randomized controlled trials of the effects on blood pressure and lipids. The American journal of clinical nutrition. 2014:ajcn. 081521. 10.3945/ajcn.113.081521 24808490

[19] Clarke R, Frost C, Collins R, Appleby P, Peto R. Dietary lipids and blood cholesterol: quantitative meta-analysis of metabolic ward studies. BMJ. 1997;314(7074):112 9006469 10.1136/bmj.314.7074.112 PMC2125600

[20] Christiansen E, Garby L. Prediction of body weight changes caused by changes in energy balance. Eur J Clin Invest. 2002;32(11):826–30. 12423323 10.1046/j.1365-2362.2002.01036.x

[21] Danaei G, Ding EL, Mozaffarian D, Taylor B, Rehm J, Murray CJL, et al The Preventable Causes of Death in the United States: Comparative Risk Assessment of Dietary, Lifestyle, and Metabolic Risk Factors. PLoS Med. 2009;6(4). 10.1371/journal.pmed.1000058 PMC2667673 19399161

[22] Prospective Studies Collaboration. Body-mass index and cause-specific mortality in 900 000 adults: collaborative analyses of 57 prospective studies. The Lancet. 2009;373(9669):1083–96. 10.1016/S0140-6736(09)60318-4 PMC2662372 19299006

[23] Norat T, Aune D, Vieira R, Chan D, D. R, Lau R. The Associations between Food, Nutrition and Physical Activity and the Risk of Pancreatic Cancer. WCRF/AICR Systematic Literature Review Continuous Update Project Report, 2011.

[24] Lewington S. Prospective studies collaboration. Age-specific relevance of usual blood pressure to vascular mortality: a meta-analysis of individual data for one million adults in 61 prospective studies (vol 360, pg 1903, 2002). Lancet. 2003;361(9362):1060-. 10.1016/s0140-6736(02)11911-8 12493255

[25] Lewington S, Whitlock G, Clarke R, Sherliker P, Emberson J, Halsey J, et al Blood cholesterol and vascular mortality by age, sex, and blood pressure: a meta-analysis of individual data from 61 prospective studies with 55000 vascular deaths. Lancet. 2007;370(9602):1829–39. 10.1016/S0140-6736(07)61778-4 18061058

[26] Forouzanfar MH, Alexander L, Anderson HR, Bachman VF, Biryukov S, Brauer M, et al Global, regional, and national comparative risk assessment of 79 behavioural, environmental and occupational, and metabolic risks or clusters of risks in 188 countries, 1990–2013: a systematic analysis for the Global Burden of Disease Study 2013. The Lancet. 2015;386:2287–323. 10.1016/S0140-6736(15)00128-2 PMC4685753 26364544

[27] Lim SS, Vos T, Flaxman AD, Danaei G, Shibuya K, Adair-Rohani H, et al A comparative risk assessment of burden of disease and injury attributable to 67 risk factors and risk factor clusters in 21 regions, 1990–2010: a systematic analysis for the Global Burden of Disease Study 2010. The lancet. 2013;380(9859):2224–60. 10.1016/S0140-6736(12)61766-8 PMC4156511 23245609

[28] NCD Risk Factor Collaboration. Worldwide trends in diabetes since 1980: a pooled analysis of 751 population-based studies with 4·4 million participants. The Lancet. 2016;387(10027):1513–30. 10.1016/S0140-6736(16)00618-8. PMC5081106 27061677

[29] Diabetes UK. The Cost of Diabetes: Report. 2014.

[30] Newton JN, Briggs AD, Murray CJ, Dicker D, Foreman KJ, Wang H, et al Changes in health in England, with analysis by English regions and areas of deprivation, 1990–2013: a systematic analysis for the Global Burden of Disease Study 2013. The Lancet. 2015;386(10010):2257–74. 10.1016/S0140-6736(15)00195-6 PMC4672153 26382241

[31] Mente A, O'Donnell M, Rangarajan S, Dagenais G, Lear S, McQueen M, et al Associations of urinary sodium excretion with cardiovascular events in individuals with and without hypertension: a pooled analysis of data from four studies. The Lancet. 2016. 10.1016/S0140-6736(16)30467-6 27216139

[32] Cogswell ME, Mugavero K, Bowman BA, Frieden TR. Dietary Sodium and Cardiovascular Disease Risk—Measurement Matters. N Engl J Med. 2016. 10.1056/NEJMsb1607161 PMC5381724 27248297

[33] World Bank. Agriculture, value added (% of GDP): The World Bank; 2016 [9 August 2016]. http://data.worldbank.org/indicator/NV.AGR.TOTL.ZS?locations=GB.

[34] FAO. Value of Agricultural Production: Food and Agriculture Organization of the United Nations Statistics Division; 2016. http://faostat3.fao.org/download/Q/QV/E.

[35] González AD, Frostell B, Carlsson-Kanyama A. Protein efficiency per unit energy and per unit greenhouse gas emissions: potential contribution of diet choices to climate change mitigation. Food Policy. 2011;36(5):562–70.

[36] Audsley E, Brander M, Chatterton JC, Murphy-Bokern D, Webster C, Williams AG. How low can we go? An assessment of greenhouse gas emissions from the UK food system and the scope reduction by 2050. UK: WWF, 2010.

[37] Scarborough P, Appleby PN, Mizdrak A, Briggs AD, Travis RC, Bradbury KE, et al Dietary greenhouse gas emissions of meat-eaters, fish-eaters, vegetarians and vegans in the UK. Climatic change. 2014;125(2):179–92. 10.1007/s10584-014-1169-1 25834298 PMC4372775

[38] Macdiarmid JI, Kyle J, Horgan GW, Loe J, Fyfe C, Johnstone A, et al Sustainable diets for the future: can we contribute to reducing greenhouse gas emissions by eating a healthy diet? The American Journal of Clinical Nutrition. 2012;96(3):632–9. 10.3945/ajcn.112.038729 22854399

[39] Perignon M, Masset G, Ferrari G, Barré T, Vieux F, Maillot M, et al How low can dietary greenhouse gas emissions be reduced without impairing nutritional adequacy, affordability and acceptability of the diet? A modelling study to guide sustainable food choices. Public Health Nutr. 2016:1–13. 10.1017/S1368980016000653 PMC10448381 27049598

[40] Wilson N, Nghiem N, Mhurchu CN, Eyles H, Baker MG, Blakely T. Foods and dietary patterns that are healthy, low-cost, and environmentally sustainable: a case study of optimization modeling for New Zealand. PLoS ONE. 2013;8(3):e59648 10.1371/journal.pone.0059648 23544082 PMC3609827

[41] Committee on Climate Change. Building a low-carbon economy—the UK’s contribution to tackling climate change. London: Committee on Climate Change, 2008.

[42] Cobiac LJ, Veerman L, Vos T. The role of cost-effectiveness analysis in developing nutrition policy. Annu Rev Nutr. 2013;33:373–93. 10.1146/annurev-nutr-071812-161133 23642205

[43] Cobiac LJ, Vos T, Veerman JL. Cost-effectiveness of interventions to reduce dietary salt intake. Heart. 2010;96(23):1920–5. 10.1136/hrt.2010.199240 21041840

[44] Wilson N, Nghiem N, Eyles H, Mhurchu CN, Shields E, Cobiac LJ, et al Modeling health gains and cost savings for ten dietary salt reduction targets. Nutr J. 2016;15(1):1. 27118548 10.1186/s12937-016-0161-1 PMC4847342

