# Supplementary material for: The Eatwell Guide: Modelling the Health Implications of Incorporating New Sugar and Fibre Guidelines
Source: PLoS One. 2016 Dec 20;11(12):e0167859. doi: 10.1371/journal.pone.0167859 (PMC5173361; doi:10.1371/journal.pone.0167859)
Supplement: S1 File — (PDF) [file pone.0167859.s001.pdf]

PRIMEtime

PRIMEtime is a new model that combines elements of the PRIME model [1], which estimates the effect of population-level changes in diet, physical activity, and alcohol and tobacco consumption on non-communicable disease mortality, with modelling methods developed in Australia to evaluate population health effects of changes in diet [2], physical activity [3], alcohol [4] and tobacco [5] consumption on both morbidity and mortality over time.

The PRIMEtime model uses proportional multi-state life table methods [6] to simulate changes in incidence, prevalence and mortality of diseases over the lifetime of the UK population. For each disease, we simulate the progression of the population through four health states: healthy, diseased, dead from the disease and dead from other causes (Figure 1). Progression through the states is based on rates of incidence, remission, case fatality and mortality.

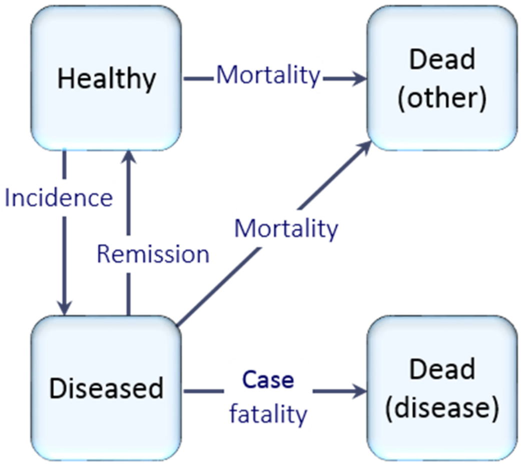

Figure 1 Modelling of diseases in PRIMEtime (after Barendregt et al. [7])

Diseases relevant to the analysis of dietary changes include coronary heart disease, stroke, type 2 diabetes, cirrhosis, and cancer of the breast, colorectum, lung, stomach, pancreas, kidney and liver.

Because diabetes is itself a risk factor for CHD and stroke, we incorporate calculations of the increased risk of CHD and stroke from the changing prevalence of diabetes using relative risks from meta-analyses of prospective population-based cohort studies of CHD and stroke incidence in men and women with diabetes [8,9].

Since some risk factors, such as body mass index (BMI), are risk factors for both diabetes and CHD and stroke, we avoid double-counting of CHD and stroke effects by reducing the relative risks of CHD and stroke from BMI. We calculate the risk reduction such that the overall fractions of CHD and stroke that are attributable to high BMI are equal to the sum of the fraction contributed directly from BMI and the fraction contributed from BMI via diabetes (Figure 2).

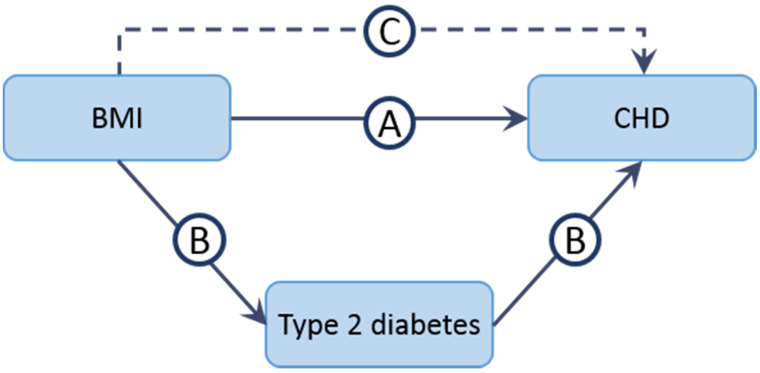

Figure 2 Using optimisation, we adjusted the relative risks for ischaemic heart disease (pathway A) such that the total fraction of ischaemic heart disease attributable to high BMI (pathway C) was equal to the contribution from BMI directly (pathway A) and the contribution from BMI via type 2 diabetes (pathway B). The same methods were used to solve for stroke risks.

The changing prevalence and mortality of diseases in the model, influences the overall number of people who remain alive in the population over time, and their quality of life associated with their disease experience.

We measure the combined effect on quantity (i.e. mortality) and quality (i.e. morbidity) of life in disability-adjusted life years (DALYs). The DALY is calculated from the number of years of life that are lived by the population, with adjustment to reflect time spent in ill-health (i.e. with 'disability' from diseases).

Model data inputs

All data inputs to the PRIMEtime model are age- and sex-specific.

- Population and mortality rates for the baseline population are extracted from the Human Mortality Database [10].
- Disease-specific incidence and case-fatality rates and baseline prevalence are derived from a combination of national registration statistics and disease-specific studies (Table 1), using DISMOD II to derive rates not explicitly reported (e.g. case fatality).
- Background trends in diseases for the UK were derived using methods developed for global burden of disease analyses [11,12].
- Disease-specific disability adjustments are determined from disability weights measured in the Global Burden of Disease study [13].

Model outputs

The PRIMEtime model simulates the impact of lifestyle changes over the lifetime of the population. Measures of output include new cases of disease or deaths from disease that are averted or delayed, changes in life expectancy, and disability-adjusted life years (DALYs) averted.

Uncertainty intervals (95%) are determined for all health outcomes using Monte Carlo analyses, based on uncertainty in model inputs (Table 2).

Table 1 Derivation of epidemiological data inputs for population health modelling.

Disease	Data and methods
CHD	Incidence of CHD estimated from incidence rates of first acute myocardial infarction (derived from Hospital Episode Statistics [14]), adjusted using the proportion of unstable angina among all coronary events in the OXVASC study [15].<br>Mortality rates from the Office of National Statistics cause-specific death registrations (number of deaths where myocardial infarction was mentioned on the death certificate).<br>Case fatality rates and baseline prevalence derived using DISMOD II.
Stroke	Incidence of first stroke estimated from the OXVASC study [15] and data from the General Practice Research Database [16].<br>Mortality rates from the Office of National Statistics cause-specific death registrations.<br>Case fatality rates and baseline prevalence derived using DISMOD II.
Type 2 diabetes	Incidence rates from the UK Clinical Practice Research Datalink [17].<br>Type 2 diabetes mortality rate ratios and prevalence estimated from the National Diabetes Audit 2011/12.<br>Case fatality rates derived using DISMOD II.
Cirrhosis	Incidence rates from a population-based cohort study linking the Clinical Practice Research Datalink and Hospital Episode Statistics [18].<br>Mortality rates from the Office of National Statistics cause-specific death registrations.<br>Case fatality rates and baseline prevalence derived using DISMOD II.
Cancer<br>– Breast<br>– Colorectum<br>– Lung<br>– Stomach<br>– Pancreas<br>– Kidney<br>– Liver	Incidence rates from Cancer Registrations Statistics, England, 2012.<br>Mortality rates from the Office of National Statistics cause-specific death registrations.<br>Case fatality rates and baseline prevalence derived using DISMOD II.

Table 2 Uncertainty assumptions for health modelling input parameters

Parameter	Risk exposure	Distributon	Mean (SD)
**RELATIVE RISKS**			
CHD	per 106 g/day Fruit	Lognormal	0.93 (0.019)
Stroke	per 106 g/day Fruit	Lognormal	0.89 (0.023)
Lung cancer	per 100 g/day Fruit	Lognormal	0.92 (0.02)
CHD	per 106 g/day Veg	Lognormal	0.89 (0.034)
Lung cancer	per 100 g/day Veg	Lognormal	0.94 (0.025)
CHD	per 10 g/day Fibre - cereal	Lognormal	0.91 (0.02)
Colorectal cancer	per 10 g/day Fibre	Lognormal	0.9 (0.034)
Breast cancer (women)	per 10 g/day Fibre	Lognormal	0.93 (0.027)
Stomach cancer	per 10 g/day Fibre	Lognormal	0.56 (0.12)
Colorectal cancer	per 100 g/day Red meat	Lognormal	1.3 (0.06)
Stomach cancer	per 100 g/day Red meat	Lognormal	1.13 (0.036)
Type 2 diabetes	per 120 g/day Red meat	Lognormal	1.2 (0.072)
Colorectal cancer	per 50 g/day Processed meat	Lognormal	1.38 (0.07)
Type 2 diabetes	per 50 g/day Processed meat	Lognormal	1.57 (0.1)

CHD			
<40 years	per -20 mmHg blood pressure	Lognormal	0.49 (0.042)
40-49 years			0.49 (0.042)
50-59 years			0.5 (0.015)
60-69 years			0.54 (0.0094)
70-79 years			0.6 (0.013)
80-89 years			0.67 (0.023)
90+ years			0.67 (0.023)
Stroke			
<40 years	per -20 mmHg blood pressure	Lognormal	0.36 (0.057)
40-49 years			0.36 (0.057)
50-59 years			0.38 (0.034)
60-69 years			0.43 (0.024)
70-79 years			0.5 (0.02)
80-89 years			0.67 (0.03)
90+ years			0.67 (0.03)
CHD			
<40 years	per -1 mmol/L total cholesterol	Lognormal	0.44 (0.034)
40-49 years			0.44 (0.034)
50-59 years			0.58 (0.022)
60-69 years			0.72 (0.018)
70-79 years			0.82 (0.015)
80-89 years			0.85 (0.021)
90+ years			0.85 (0.021)
Stroke			
<40 years	per -1 mmol/L total cholesterol	Lognormal	0.9 (0.037)
40-49 years			0.9 (0.037)
50-59 years			0.9 (0.037)
60-69 years			1.02 (0.027)
70-79 years			1.04 (0.025)
80-89 years			1.06 (0.031)
90+ years			1.06 (0.031)
CHD			
35-59 years	per 5 kg/m2 BMI	Lognormal	1.5 (0.039)
60-69 years			1.4 (0.031)
70-79 years			1.31 (0.033)
80-89 years			1.3 (0.055)
Stroke			
35-59 years	per 5 kg/m2 BMI	Lognormal	1.76 (0.075)
60-69 years			1.49 (0.056)
70-79 years			1.33 (0.056)
80-89 years			1.1 (0.083)
Diabetes			
15-25 units	per 5 kg/m2 BMI	Lognormal	0.96 (0.25)
25-50 units			2.16 (0.067)
Cirrhosis			
15-25 units	per 5 kg/m2 BMI	Lognormal	0.73 (0.16)
25-50 units			1.79 (0.077)
Colorectal cancer			
Men	per 5 kg/m2 BMI	Lognormal	1.24 (0.016)
Women			1.09 (0.019)
Kidney cancer			
Men	per 5 kg/m2 BMI	Lognormal	1.24 (0.039)
Women			1.34 (0.034)
Liver cancer	per 5 kg/m2 BMI	Lognormal	1.47 (0.078)
Breast cancer (women, 60+)	per 5 kg/m2 BMI	Lognormal	1.12 (0.018)

Pancreas cancer	per 5 kg/m2 BMI	Lognormal	1.1 (0.016)
**INTERMEDIATE VARIABLES**			
BP	per 100mmol/24hr sodium	Normal	5.8 (1.71)
TC	per 1% energy total fat	Normal	0.02 (0.005)
TC	per 1% energy saturated fat	Normal	0.052 (0.003)
TC	per 1% energy MUFA	Normal	0.005 (0.003)
TC	per 1% energy PUFA	Normal	-0.026 (0.004)
TC	per 1 g/day dietary cholesterol	Normal	0.0007 (0.0001)
TC	per -18.9% energy added sugar	Normal	-0.23 (0.056)
CHD			
Men	Diabetes prevalence	Lognormal	1.85 (0.063)
Women			2.63 (0.076)
Stroke			
Men	Diabetes prevalence	Lognormal	1.83 (0.067)
Women			2.28 (0.085)
**THEORETICAL MINIMUM RISK**			
BMI (kg/m2)	–	Normal	21 (1)
Blood pressure (mmHg)	–	Normal	115 (6)
Total cholesterol (mmol/L)	–	Normal	3.8 (0.6)
Vegetable intake (g/day)	–	Normal	400 (30)
Fruit intake (g/day)	–	Normal	300 (30)
Fibre intake (g/day)	–	Normal	30 (3)
Red meat intake (g/day)	–	Normal	14.3 (1.43)
Processed meat intake (g/day)	–	-	0
**MEDIATION FACTORS**			
Ischemic stroke mediation			
BMI via Blood pressure	–	Normal	0.65 (0.04)
Fruit intake via Blood pressure			0.42 (0.17)
Vegetable intake via Blood pressure			0.54 (0.2)
Fruit intake via Total cholesterol			0.027 (0.017)
Vegetable intake via Total cholesterol			0.047 (0.026)
Ischemic heart disease mediation			
BMI via Blood pressure	–	Normal	0.31 (0.016)
Fruit intake via Blood pressure			0.39 (0.15)
Vegetable intake via Blood pressure			0.47 (0.21)
Fruit intake via Total cholesterol			0.008 (0.0057)
Vegetable intake via Total cholesterol			0.012 (0.01)

References

1. Scarborough P, Harrington RA, Mizdrak A, Zhou LM, Doherty A (2014) The Preventable Risk Integrated ModEl and Its Use to Estimate the Health Impact of Public Health Policy Scenarios. Scientifica 2014.
2. Cobiac LJ, Vos T, Veerman JL (2010) Cost-effectiveness of interventions to promote fruit and vegetable consumption. PLoS One 5: e14148.
3. Cobiac LJ, Vos T, Barendregt JJ (2009) Cost-effectiveness of interventions to promote physical activity: a modelling study. PLoS Med 6: e1000110.

4. Cobiac LJ, Vos T, Doran C, Wallace A (2009) Cost-effectiveness of interventions to prevent alcohol-related disease and injury in Australia. Addiction 104: 1646-1655.
5. Blakely T, Cobiac LJ, Cleghorn CL, Pearson AL, van der Deen FS, et al. (2015) Health, health inequality, and cost impacts of annual increases in tobacco tax: Multistate life table modeling in New Zealand. PLoS Medicine 12.
6. Barendregt JJ, Van Oortmarssen GJ, Van Hout BA, Van Den Bosch JM, Bonneux L (1998) Coping with multiple morbidity in a life table. Mathematical Population Studies 7: 29-49.
7. Barendregt J, Van Oortmarssen G, Vos T, Murray C (2003) A generic model for the assessment of disease epidemiology: the computational basis for DisMod II. Population Health Metrics 1: 4.
8. Peters SA, Huxley RR, Woodward M (2014) Diabetes as a risk factor for stroke in women compared with men: a systematic review and meta-analysis of 64 cohorts, including 775 385 individuals and 12 539 strokes. The Lancet 383: 1973-1980.
9. Peters SA, Huxley RR, Woodward M (2014) Diabetes as risk factor for incident coronary heart disease in women compared with men: a systematic review and meta-analysis of 64 cohorts including 858,507 individuals and 28,203 coronary events. Diabetologia 57: 1542-1551.
10. University of California Berkeley (USA), Max Planck Institute for Demographic Research (Germany) Human Mortality Database.
11. Mathers CD, Loncar D (2006) Projections of global mortality and burden of disease from 2002 to 2030. PLoS medicine 3: e442.
12. Salomon JA, Murray CJ (2002) The epidemiologic transition revisited: compositional models for causes of death by age and sex. Population and Development Review 28: 205-228.
13. Lim SS, Vos T, Flaxman AD, Danaei G, Shibuya K, et al. (2013) A comparative risk assessment of burden of disease and injury attributable to 67 risk factors and risk factor clusters in 21 regions, 1990–2010: a systematic analysis for the Global Burden of Disease Study 2010. The lancet 380: 2224-2260.
14. Smolina K, Wright FL, Rayner M, Goldacre MJ (2012) Incidence and 30-day case fatality for acute myocardial infarction in England in 2010: national-linked database study. The European Journal of Public Health 22: 848-853.
15. Rothwell P, Coull A, Silver L, Fairhead J, Giles M, et al. (2005) Population-based study of event-rate, incidence, case fatality, and mortality for all acute vascular events in all arterial territories (Oxford Vascular Study). The Lancet 366: 1773-1783.
16. Lee S, Shafe AC, Cowie MR (2011) UK stroke incidence, mortality and cardiovascular risk management 1999–2008: time-trend analysis from the General Practice Research Database. BMJ open 1: e000269.
17. Holden S, Barnett AH, Peters JR, Jenkins-Jones S, Poole CD, et al. (2013) The incidence of type 2 diabetes in the United Kingdom from 1991 to 2010. Diabetes, Obesity and Metabolism 15: 844-852.
18. Ratib S, West J, Crooks CJ, Fleming KM (2014) Diagnosis of liver cirrhosis in England, a cohort study, 1998–2009: a comparison with cancer. The American journal of gastroenterology 109: 190-198.
